# Ten Letters, with Answers

**Published:** 1873-07

**Authors:** 


### Our Correspondence.

### Letters Exposing Humbugs and Swindlers,

### With other Communications of Interest to the People.

Hiesville, Ky., June 14, 1873.

*Editor Bistoury* :—I remember reading several valuable receipts in former numbers of the Bistoury, for the diarrhœa common to children and adults during the hot season. I had one formula prepared last season, and found it excellent, having had numerous occasions to use it in my own and neighbor's families. I have lost the receipt and some one has been thoughtless enough to carry off my Bistoury. You will greatly oblige me by giving us another formula, for the same complaint, in the July number.

Mrs. H.

A very excellent formula for diarrhœa during the heated season, is the following :

Take of—
Tincture of Capsicum,
Tincture of Camphor,
Tincture of Rhubarb, of each 1 ounce;
Tincture of Opium, half an ounce.

Mix, and give a child four years old, six drops on a lump of sugar every two hours, until the pain is arrested.

The same medicine in larger doses will be found useful and timely in the pains and diarrhœas of older persons.

---

Penn Yan, N. Y., May 16, 1873.

Editor Bistoury :

You seem to be "good for" almost every complaint, tell me, pray, what shall I do for a lump or tumor, the size of a hen's egg, upon the top of my head ? It has been growing for ten years, is soft to the touch, has never been painful, but is not particularly ornamental.

J. P. R.

It is probably an encysted tumor, which can be removed by a surgeon. The operation is attended with but little pain and no danger. Have it out.

---

Harrisburg, Pa., May 20, 1873.

Editor Bistoury :

My wife has been to Philadelphia and had a cancer plaster applied to her breast, by a celebrated cancer doctor of that city. Her breast had a small lump in it about the size of a pullet's egg, but was not sore until this plaster was placed on it to eat it out. The doctor sent her home saying the cancer was all out and that the wound would soon fill up. It does *not* heal however, the breast is very much swollen and very painful, so that we are becoming alarmed about it. I saw your BISTOURY at the house of a friend, and thought I would write you for advice. Now, what shall we do? L. B.

We are much afraid that you have involved the case in serious difficulty. A "cancer plaster" is the very worst application you could have made. We have seen very many victims to this abominable process,—many innocent tumors, that never would have caused trouble, converted into malignant growths, and producing death, through the application of these escharotic plasters. We are fearful that the case is beyond the reach of surgery, but would advise you to consult some competent surgeon, one who deals with the knife and not with an irritating plaster.

---

HARTFORD, Kansas, May 28, 1873.

DR. UP DE GRAFF:

Is John B. Ogden, of Station D, Bible House, New York, and who advertises a free formula, a reliable man? Does he mean to benefit the afflicted, without charge, as he claims in his advertisement? R.

You are evidently an innocent, one not posted in the tricks and trades of the sharpers of New York. Read more, my friend, and place no confidence in J. B. O. or any of his ilk,—he's a villianous fraud, many times exposed in the BISTOURY.

---

KANSAS CITY, Mo., June 2, 1862.

EDITOR BISTOURY:

What would you use for indurated and enlarged tonsils in children? Where can I procure a good tonsillitome?

J. T. B., M. D.

We are in the habit of using a saturated solution of tannin in tincture of iodine, to enlarged tonsils, with excellent results. Paint the glands twice a day, and gargle with cold water ten minutes after the application.

Write to Geo. Tieman & Co., 67 Chatham Street, New York, for the instrument. Think they have a branch in St. Louis.

---

TRUMANSBURG, N. Y., June 14, 1873.

EDITOR BISTOURY:

What would you do to drive out measles? J.

Pour boiling water on them. If that don't start them, try non-explosive oil or plastic slate roofing. If they can stand *that*, lay for them with a breech-loader and No. 4 shot.

The best plan, though, is to let them alone, and not drive them at all.

---

JACKSONVILLE, Fla., June 1, 1873.

DEAR DOCTOR:

Have you professionals sent *all* your invalids to the St. Johns, or are there really a sufficient number left at home to pay the current expenses of your respective families?

I haven't seen a well-looking face, upon sound shoulders, since my sojourn here.—Cough, sneeze, spit, spew are the familiar noises to be seen, heard and smelt on all sides. Jacksonville is an immense open-air hospital for the congregation of the sick of the north. One don't feel comfortable here unless he has something the matter with him. You must be sick to be appreciated. Well folks have no business here, save to take care of and amuse the sick ones. Remain here a day, and be you ever so well, you become sick through sympathy. We go for allegators to-day, they being the only sound inhabitants of the St. Johns. We can't afford to allow them to bask in the sun, feeling so confoundedly comfortable, when everybody else is sick. We are "going for 'em" with rifles, just to make them feel like other folks.

Yours, as ever,

PATIENT.

---

KINGSBURY, Ind., April 18, 1873.

THE BISTOURY, Elmira, N. Y.:

The BISTOURY for April, has to date failed to appear. You know I told you I did not want the January number, but to let my subscription commence April 1st. If you have forgot to send it, I will forgive, if you will send it, but if you propose to swindle, I want to know it and then I will know who I am dealing with. Send the April number *as soon as possible* and oblige,

Yours, truly,

WM. E. HEWSON.

Mr. H. is evidently one of the impatient and suspecting sort of individuals, who sees a stupendous swindle behind every dilatory half dollar. We hope his number of the BISTOURY arrived in due season, and that he lost but few sleepless nights before he discovered that he was not "swindled" after all.

New Providence, Ala., May 17, 1873.
Dr. Up de Graff :

Enclosed, please find 50 cents, for which you will send Bistoury for one year.

I have noticed your answers to correspondents, and if it will not be a trespass on your time, please answer this query : I am afflicted with a sick headache, and have been for twenty-five years. Nothing seems to relieve me. If you will give me your advice, I will ever remember your kindness.

Respectfully,
H. D. C.

Sick-headache is the most common morbid affection of the nervous system, arising from some form of indigestion. The state or condition of the brain depends much upon that of the stomach. No two cases of sick-headache require practically the same kind of treatment. Therefore no directions can be given that will apply in all cases. Overloading the stomach —cramming it with more food than it is capable of caring for—indigestion of meat, vegetables, milk, fat, etc., all cause certain forms of headache. To prescribe for you understandingly would require a careful examination into the condition of your stomach, ascertain what its capabilities are for disposing of certain qualities of food, and discarding all kinds that prove to be unacceptable to it. Attention to diet, suitable medication or hygenic advice for relief of constipation, and the administration of tonics are indicated. Seek the advice of a competent physician and follow his directions.

---

New Providence, Ala., May, 19, 1873.
Dr. Up de Graff:

I have a son who is a source of anxiety to me. I have tried several physicians, and all kinds of medicine, but to no purpose, from the simple fact we do not know what we are treating, or the nature of his disease. Several of my friends have advised me to write to you, to get your ideas of his disease. The symptoms are as follows:

He is about five years old, apparently in fine health, robust, and well muscled. When about a year old he would often have fevers, and every fever would be accompanied with a fit or spasm. He continued to have them regularly, until about twelve months ago, when he would have something like a fit without any fever. They have increased on him till now he has them as often as twice a day.—They last him but a few moments. While they are on him he cannot speak. If he has a spell in the afternoon he is sure to go to sleep, and will sleep soundly all night. He has a strong desire for tobacco, and will smoke more than any person I ever saw. Some two months ago I stopped him from smoking. He now chews tobacco all the time. I have never known of his being the least sick from tobacco. He has a ravenous appetite, and I have known him to eat as many as ten eggs at one meal, withother food. Excuse length, as I wanted you fully to understand the case.

I will be ever grateful if you will answer the above, and give me your opinion of the disease, with remedy.

Very respectfully,
W. E. G. H.

Your child has epilepsy, but from what cause does not clearly appear in your description of his case. Probably caused from some abnormal condition of his digestive apparatus. Send him to some hygenic establishment where his appetite can be controlled. Home is not the place for him, evidently. Large doses of bromide of potassium would be a suitable substitute for the tobacco, and would doubtless satisfy his craving for stimulants.—He should be placed under the care of a competent physician in some institution where such cases are specially treated, when, we doubt not, his malady could be cured within a reasonable time.

---

Dr. G. D. M.—*Hartford, Kan.*—The extra dollar was received.

---

E. H.—*Lockhart, Texas.*—Your communication containing five dollars, and which was mailed as a registered letter, Feb. 20, 1872, has finally come to hand. We have ordered the journals to commence with the July numbers.

---

Dr. S. P.—*Covington, Ky.*—An answer to your query will be found in the Medical Items and News. Address Geo. Tiemann & Co., 67 Chatham St., N. Y.

---

Dr. A. A. D.—*Humboldt, Tenn.*—Use "india ink," rubbedin water, to consistence of cream. Spread upon the opake spot, and prick it into the cornea with three or four fine needles bound to a stick. Repeat the operation as often as may be necessary to secure the required hue.

